# Is Purchase Behavior Different for Consumers with Long COVID?

**DOI:** 10.3390/ijerph192416658

**Published:** 2022-12-11

**Authors:** Alicia Blanco-Gonzalez, Gabriel Cachón-Rodríguez, Cristina Del-Castillo-Feito, Ana Cruz-Suarez

**Affiliations:** Business Economics Department, Rey Juan Carlos University, 28933 Móstoles, Spain

**Keywords:** COVID-19, long COVID, purchase behavior, legitimacy, uncertainty

## Abstract

COVID-19 has generated an uncertain environment, which has motivated changes in consumers’ behavior globally. However, previous studies have not clarified if these effects are equally strong throughout the population. In this research, we want to analyze if there are behavioral differences between long-COVID consumers and others. For this purpose, we analyzed a sample of 522 consumers divided into three groups depending on their type of exposure to the disease: those with long COVID; ones that had recovered from COVID-19; and those that had never had COVID-19. The results show that the effect that COVID-19 has on purchase behavior differs depending on the type of exposure to the disease. In fact, those with long COVID experienced more pleasure when purchasing than other people, but they needed higher trust levels in the enterprises to purchase from them, since that reduces their perception of uncertainty. Furthermore, for long-COVID individuals, an organization’s legitimacy level is even more important than for other consumer groups with less contact with the disease.

## 1. Introduction

The COVID-19 pandemic has generated a great socioeconomic impact on the global population. From a social perspective, more than six million deaths occurred, and there was an increase in mental health diseases by 25% [1]. Economically, it has had a negative impact on the GDP of every economy, resulting in the bankruptcy of many enterprises. The socioeconomic consequences have been enormous. The changes in consumers’ behavior have been among the most relevant [2].

Consumers have faced confinement, hygiene, security, and social-distancing measures [3]; scarcity of food and essential products; limited distribution of critical goods; cancellation of services; and increases in unemployment due to firms’ bankruptcy [2]. In this context, sales have increased for specific types of products: food and hygiene products [4], prosumer behaviors, or do-it-yourself products [5,6]. However, there was a decrease in sales for other products, for example, clothes and leisure activities [4,6].

In general, the crisis generated by COVID-19 not only changed what was bought, but also how it was bought [2]. These types of changes in consumer behavior have been analyzed in academical studies about consumer behavior during disruptive circumstances, such as natural disasters, terrorism, and epidemic breakouts [7]. Cruz-Cárdenas et al. determined that consumers around the world develop impulsive behaviors and product-storage behavior. Product-storage behavior is a type of behavior that develops during panic reactions [8] and it increases impulsive purchases [9]. In fact, it has been proven that COVID-19 influenced consumers’ living and thinking patterns, resulting in one of the variables that has had the strongest effect on impulsive purchase behavior [10]. Other factors that have impacted in consumer behavior are digitalization [11] and the problems of mental health [12]. The increase in online purchases at the expense of purchases in physical stores was confirmed. This is, in part, because in some situations, people prefer virtual interactions instead of physical interactions [11]. Moreover, when a consumer has mental health problems (anxiety or stress), he or she often prefers to buy from online platforms to avoid social interactions.

The scientific community agrees that experiences that affect the population, such as COVID-19, impact consumer behavior. Therefore, managers must adapt their business and commercial strategies. The question is whether this impact is the same for the entire population. We considered whether, based on the experience with the virus, the behavior of consumers differs, and ultimately, whether it is necessary to adapt business strategies depending on the type of consumer. We have found that disruptive events, especially disease outbreaks, can influence the consumer behavior of those individuals who have suffered from the disease (direct effect) and on the behavior of people who have not suffered it, for example, due to fearing contagion (indirect effect). 

We consider that, as consumer behavior can vary depending on the uncertain situation under consideration [12,13], COVID-19 can generate purchase behaviors that differ depending on the exposure to the disease: those that suffer permanent consequences (long COVID); those that have suffered and recovered without after effects; and those that have never been infected. Following the World Health Organization, persistent or long COVID is a syndrome characterized by the persistence of symptoms of COVID-19 weeks or months after the initial infection, or by the appearance of symptoms after a time without them (breathing problems, headaches, vomiting, extreme exhaustion, etc.). Its appearance is not related to the severity of the initial infection, so it can affect both mild and severe hospitalized patients. In addition, it affects people of any age and has a high impact on one’s quality of life, work, and social environment.

The main objective of this research was to analyze the significant differences caused by COVID-19 in purchase behavior and its antecedents, depending on the type of exposure to the disease. The aims of this study were: (1) to identify different clusters in the marketing, which were generated by the virus; (2) to determine if key variables, such as anxiety, a perception of uncertainty, company legitimacy, and emotional regulation, impact consumer behavior in a health crisis; and (3) to analyze whether long COVID is a problem such that authorities and companies must adapt their policies and design strategies to this segment of the population.

In the next section, we show the theoretical framework for the research. We describe the relationship between purchasing intention and its antecedents. We will focus on the following antecedents: uncertainty, legitimacy, anxiety, and emotional regulation. The theoretical description of the relationship between purchase behavior and its antecedents supports the proposal of four hypotheses to test in this investigation. After the theoretical analysis, we describe the applied methodology and the results. Finally, the main conclusions are presented. 

## 2. Theoretical Framework

**Figure 1:**
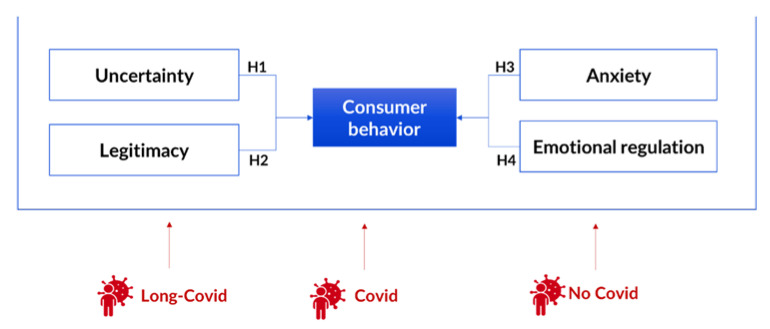
Theorical model and hypothesis. Source: own elaboration.

In this section, following academical papers [3,4,7], we describe some of the most important purchasing behaviors in crises. We argue for the possibility of the type of exposure to COVID-19 causing changes in purchasing behavior, since the purchasing behavior of those who have permanent/long-term consequences (long COVID), those that have recovered without after effects (COVID-19), and those who have never been infected (no COVID-19) might differ (Figure 1).

### 2.1. External Antecedents: Uncertainty and Legitimacy

Uncertain scenarios reflect situations with low security and trust levels that impact on consumer behavior [12]. These scenarios have been widely analyzed in the literature, especially in research papers about consumer behavior [14]. For example, during the COVID-19 pandemic, many supermarkets ran out of toilet paper, hand sanitizer, and canned food products [6]; further, the drop-out intention rates of students increased [15]. Perceived severity, in other words, how likely individuals perceive themselves to be at risk, increases the intention to make unusual purchases [9]. In fact, Islam et al. (2021) demonstrated that during the pandemic, perceived arousal had a positive impact on impulse buying behavior and on obsessive-compulsive buying [8]. Milton (2022) showed various evidence supporting that uncertain situations generate negative, neutral, and/or positive changes in consumer behavior and purchase intention [16]. 

In general terms, consumers’ risk perceptions have a negative influence on purchase behavior [17,18]. These behaviors are developed with the purpose of reducing risk and have a direct and indirect impact on consumer behavior [19]. When facing risk situations, people tend to choose less uncertain options [20]. During the pandemic, several trends were observed: for example, meat consumers in Brazil are more likely to purchase when purchase uncertainty is reduced, for example, through the application of tracking systems [21].

Nevertheless, the effect of uncertainty over behavior is not always the same. Individuals reach in different ways when facing uncertainty situations, depending on their cognitive approaches [13]. During the COVID-19 crisis, men and women used different mindsets when evaluating organizations’ legitimacy. While cognitive evaluation criteria predominate in men, pragmatic evaluation criteria were more relevant in women, under higher uncertainty [12]. In this context, there is a large amount of literature regarding differences in behavior for men and woman in risky situations [22,23]. Emotional factors are also capable of generating changes in consumers’ behavior. For example, variables, such as anxiety, demonstrated their influence over consumers behavior during the pandemic [24]. Consumers with higher levels of anxiety buy more supplies than those with less anxiety [25]. Therefore, we propose the following hypothesis:

**Hypothesis 1.**  *Uncertainty influences purchase behavior depending on the type of exposure to COVID-19 (long COVID, COVID-19, No COVID-19).*

Under uncertain situations and global crises, demonstrating conformity to socially shared norms and values increases the probabilities of organizational survival [26]. According to the Institutional Theory, stakeholders’ perceptions and assessments will be positive when institutions behave in a socially accepted manner in society [27], since individuals connect with and support corporations with which they share values and beliefs [28].

Social acceptance is highly connected with organizational legitimacy. Legitimacy has been defined as the perception of an organization’s appropriateness within a social system with specific values, beliefs, and rules [29]. Without being socially perceived as legitimate, an organization will not be able to operate in the market [30]. 

Legitimated entities are regularly more successful than those without legitimacy [31] since they will have better access to critical resources for their activity [32]. They will also improve their credibility and trust levels in front of society [27,33]. In fact, a clear relationship between holding high legitimacy levels and better organizational performance has been identified [34].

Since legitimacy is highly connected with the ability that an enterprise has to comply with customer’s values and needs [35], its correct management can influence consumer behavior, resulting in higher purchase intention levels [36,37,38]. Positive legitimacy perceptions will grant consumers’ support in the short term through the increase in purchase intention [39], which could also translate into long-term results through their purchase repetition and loyalty achievement [40]. Based on this analysis, the following hypothesis is proposed:

**Hypothesis 2.**  *Perceived legitimacy influences purchase behavior depending on the type of exposure to COVID-19 (long COVID, COVID-19, No COVID-19).*

### 2.2. Internal Antecedent: Anxiety and Emotional Regulation

Epidemiological evidence has highlighted anxiety as an emotional state associated with negative emotional states, such as depression, fear, and rage, emanating from the uncertainty associated with COVID-19 [41,42]. The anxiety concept is related to an imbalance resulting from concerns, tension, and fear about what is about to come [43,44,45]. It represents a transitional psychological state, presenting physical excitation, tension, compression, and fear for what could happen [46]. Within the emotions theory [47], the concept “emotions” was defined as “organized cognitive-motivational-relational configurations which state changes with changes in the relationship person-environment depending on how they are perceived and evaluated”. 

Scherer [44] defines an emotion as an episode of organic changes in cognitive, attitudinal, and behavioral components experienced by an individual [48]. When examining anxiety, the internal and external stimuli evaluation identifies a threat, even though this threat is not real [49]. Anxiety might not be objective and the negative feeling produced by it too intensive considering reality [50]. Thus, anxiety is the result of a perceived threat [49,51], which negatively affects the person’s happiness, his/her self-esteem, and capacity of processing the experienced information in a realistic manner [52,53].

In a decision-making process, different theories can relate anxiety with purchase intention. The Expectancy Theory is a motivational perceptual theory based on human behavior [54]. This theory suggests that a person tends to behave in a specific manner according to the award expectancy that he/she will receive when developing an action [55]. From this approach, anxiety is the result of a negative stimulus that impedes a relaxed behavior and that leads consumers to avoid certain behaviors [56]. In this sense, anxiety represents fear associated with future consequences, which discourages purchase intention [57]. 

The Reactance Theory considers that people believe that they have certain freedoms and when these liberties are challenged, a psychological reactance appears [58]. The reactance is a motivational state aimed to restore or ensure freedom [59]. From this theory’s point of view, anxiety is understood as a limiting factor, which affects individual freedom and consumer recovery [56,58]. These theories contribute to the understanding and elaboration of a valid theoretical framework regarding the psychological effects over purchase intention. 

Regarding purchase behavior, previous studies have linked anxiety with threats or fear to future consequences related to purchases and lack of control over expected results [60]. Consumers with high anxiety levels usually create exaggerated negative perceptions [61,62] and those assessments play a critical role in their decision-making process, even when these decisions are irrelevant [63]. Celik [64] demonstrated how anxiety was one of the main factors affecting purchase processes. Different authors have considered anxiety as a factor with a negative and significant impact on one of the purchase intention antecedents, which is usage intention [65,66]. Previous studies have suggested that COVID-19 has generated a psychological effect, with consumers motivating an increase in their anxiety level [67]. 

Other authors have connected the anxiety negative effect with panic purchase processes [56]. Therefore, consumers with high anxiety levels are more likely to behave in a risk-averse mode and to evaluate external stimuli as imminent dangers [61]. Anxiety is a purchase process psychological factor related to health [3], which affects consumption decisions and leads to an avoidance behavior [68]. 

Nevertheless, in a situation, such as the one generated by COVID-19, fear and anxiety were able to limit cognitive and attention elements, in a way that only immediate needs were considered [25]. People that experience negative emotions can consume products with a hedonic benefit with the aim of mitigating or reducing the negative feelings. In fact, consumption is one of the applied strategies for people who experience negative emotions to achieve a positive reinforcement [69]. Under these circumstances, anxiety can have adaptive characteristics since individuals are motivated to protect themselves and consumers feel the need to buy based on their crisis perceptions. Thus, the anxiety generated by COVID-19 caused an increase in big amounts of purchases with the objective of being more protected from a potential threat.

**Hypothesis 3.**  *The anxiety level influences purchase behavior depending on the type of exposure to COVID-19 (long COVID, COVID-19, No COVID-19).*

Within the psychology literature, emotional regulation has been defined as the process to cushion, intensify, or simply maintain an existing emotion [70]. From the perspective of the Hedonic Contingency Theory, the concept of emotional regulation is associated with a mental process, which tries to adjust positive emotions to maintain of intensify positive feelings [71]. Consumption emotional regulation is defined as product or service consumption or purchase with the purpose of relieving, repairing, or managing an emotion in the short run [72]. Previous literature has identified that the purchase process is related with the process of lowering negative emotions, such as fear or anxiety [47]. However, when facing negative emotional states, consumers try to maintain positive emotional states in an active manner [69,73]. 

Previous research papers have found that emotional states result in different assessments of an individual’s environment and, therefore, can have implications for consumers’ choices and decision-making processes [61]. Fredrickson et al. [74] developed a study about emotions and the cardiovascular effects, where they discovered that funny movies that generate satisfaction produced faster cardiovascular recoveries than neutral or sad movies. These findings suggest that positive emotions have the capacity to reduce or even eliminate negative emotions’ effects. Wegener et al. (1995) discovered that individuals with happy emotional states pay more attention to communication messages for their hedonic consequences than people with sad emotional states [75]. Garg et al. (2007) demonstrated that consumers are willing to consume wider amounts of a product when they are happy than when they are sad [76]. Other studies have shown that people facing situations that generate negative emotions (for example, bad day at work or bad family relationships) can increase their consumption as a mechanism to improve their emotional state [77]. In more extreme situations, such as COVID-19, people increase the level of purchases of hedonic products, such as alcoholic beverages, candy, or take-away food, to face the critical event of the pandemic [25].

**Hypothesis 4.**  *Emotional regulation influences purchase behavior depending on the type of exposure to COVID-19 (long COVID, COVID-19, No COVID-19).*

## 3. Sample and Methodology

To test the proposed hypotheses, an empirical study with a sample of 521 people was developed, including: 121 individuals with long COVID (long-COVID sample suffers symptoms of COVID-19 weeks or months after the initial infection (breathing problems, headaches, vomiting, extreme exhaustion, etc.) and long COVID impacts one’s quality of life, work, and social environment); 200 people who had recovered from COVID-19 without after effects (these people suffered mildly or severely from the disease but are recovered and have no physical or psychological sequelae of the disease); and 200 people who had never been infected (they are people who have never been diagnosed with COVID-19. In general, they do not know why they have not suffered from the disease, even though they are surrounded by many cases of the disease and have sometimes even lived with COVID-19 patients). To gather the data, we contacted a market-research-specialized company who distributed the online surveys and gathered the data during June 2022 in Spain. To identify which group they belonged to, they were asked a series of questions about when and how they suffered from the disease and, if they had sequelae, what they were. In order to correctly identify the study subjects, we worked together with experts in the field of health.

In addition, control variables have been incorporated to verify that the sample and the results are adequate. On the one hand, variables related to COVID-19 were incorporated: when they suffered the disease, if they have been hospitalized due to this virus and the symptoms of long COVID identified by World Health Organization (vomiting, dyspnea, headache, diarrhea, diarrhea, skin problems, or memory loss, among others). On the other hand, control variables related to the sociodemographic and socioeconomic characteristics of the respondents (age, gender, population, educational level, and income level) were incorporated.

The online survey included items based on the literature on the topic to measure the considered variables (Table 1). To measure purchase behavior, we included 4 items based on [25,69]. Regarding external factors (uncertainty and legitimacy), we included 3 and 7 items, respectively, based on [3,20]. To measure internal factors (anxiety and emotional regulation), we included 8 and 1 item, respectively, based on the studies developed by [9,25].

The applied methodology for the treatment of the data is structured in the following order: first, we analyze the reliability and validity of the measurement scales through an Exploratory Factor Analysis; second, we analyze if there are significant differences between the samples through the variance analysis (ANOVA analysis), and third, we evaluate the hypotheses through a regression analysis. The applied software to analyze the results was SPSS version 21.

The regression analysis verifies the impact of independent variables (uncertainty, legitimacy, anxiety, and emotional regulation) on dependent variable (purchase intention). The first test analyzed Fisher’s F statistic (the one obtained from an analysis of variance) that determines if the parameters are equal or different from “zero”. This value is significant when *p* < 0.05. The second test is the evaluation of R^2^ or coefficient of determination that determines the fit of the model, or in other words, how much of the dependent variable is explained by the independent variables. Finally, the significance of the hypothesis is evaluated through a *p*-value < 0.05.

## 4. Results

The evaluation of the hypotheses is developed in three phases. First, we develop an Exploratory Factor Analysis to validate if the measurement scales are appropriate to develop a causal analysis. As shown in Table 2, the results from the factorial analysis indicate that the scales are adequate, the extracted loadings are higher than 0.6 in every item, the Cronbach Alpha is over 0.70, and the extracted variance is higher than 60%.

Second, we analyze if there are significant differences between the results from the three considered groups. Table 3 shows the results on the analysis of variance (ANOVA), which confirms that there are significant differences between groups, for all the considered variables. These results highlight the need to develop an individual regression analysis for each of the considered groups to determine which specific factors influence their purchase intention. 

Third, we analyze the cause–effect relationship between purchase intention and each of its antecedents, depending on the type of exposure to COVID-19. Considering the sample with long COVID, the regression explains 42.5% and the antecedents that influence their purchase intention are: emotional regulation (0.508); uncertainty perception (0.154); and legitimacy assessments (0.144). Regarding those people who have never been infected with COVID-19, the regression explains 34.5% of their behavior, with the following factors determining their purchase intention: emotional regulation (0.392); uncertainty perception (0.237); and anxiety level (0.224). Finally, regarding those that have recovered from COVID-19 without after effects, the regression explains 20.7%, with emotional regulation (0.262) and anxiety (0.249) as the main purchase intention determinants. The results are presented in the following table (Table 4).

## 5. Conclusions

COVID-19 has not only influenced purchase behavior worldwide, but this effect has been different depending on the type of exposure to the disease (long COVID, COVID-19, no COVID-19) and this demonstrates that everybody should understand the relevance of the consequences of this disease. In this research, we want to help inform governments and organizations about long COVID, because long COVID has a high impact on quality of life, work, and social environment. For this, in this research, we identify that there are differences in the purchase behavior of consumers with long COVID and other consumers. 

The results confirm this approach, since all four proposed hypotheses were supported, demonstrating that external factors (H1 and H2) and internal factors (H3 and H4) impact consumer behavior depending on the type of exposure to COVID-19 (long COVID, COVID-19, no COVID-19). After all, consumer behavior reflects lived experiences. Not only living through a health pandemic, but also our relationship with the virus has generated a different state of mind, uncertainty management, or perceptions of what companies should do. Likewise, considering the percentage of the population that has suffered from COVID-19 and that approximately 10% of those who have contracted the disease will suffer from long COVID, it is essential that those responsible for analyzing consumer behavior take these factors into account.

The results suggest that uncertainty (H1) is a variable without an impact in the purchase behavior of those individuals that have recovered from COVID-19. For these people, COVID-19 is not perceived as risky since they have already recovered from it. However, this construct does have an impact on other people, especially in those individuals who have not yet been infected. Legitimacy (H2) has become a critical variable for people with long COVID. In fact, this antecedent only has an effect on purchase intention when considering people with long COVID. This result indicates that those individuals who experience after effects from the disease need to feel stronger trust and reliability perceptions from those companies from which they are going to buy products or services. Therefore, the importance of legitimacy for long-COVID consumers is higher. The reduced importance this groups gives to legitimacy, the greater its purchase intention will be. Anxiety (H3) impacts both individuals with no COVID-19 and those recovered from COVID-19 but does not influence long-COVID consumers. This result suggests that having after effects from COVID-19 creates a complex state that covers the effect that anxiety can have on purchase intention. Finally, emotional regulation (H4) is an antecedent present for every segment. We observe that deriving pleasure from purchasing is higher for long-COVID consumers. This can be explained due to the possibility of escaping from the after effects of COVID-19 while purchasing.

The results involve important implications for companies and governments. It was confirmed that purchase behavior antecedents influence in a different manner for long-COVID consumers. Therefore, companies should take into consideration this situation for their relationships with this specific segment. Those who suffer long COVID want to experience more pleasure when they buy, but they need to perceive higher trust levels towards companies to purchase from them, since trust reduces uncertainty perceptions. Enterprises can build trust through the development of strategies to increase their legitimacy [32].

This study is not without limitations, especially related to the size of the sample and the fact that the study was carried out in Spain. Likewise, another limitation is that it asks about behavior in general and analyzing a specific behavior, for example, analyzing how they behave when they buy in the supermarket, which would give us a particular vision of these changes.

This research has sparked our interest in investigating how COVID-19 impacts people’s lives and their behavior. We consider it very important to continue analyzing the effects of long COVID on consumer behavior and to inform the population, governments, and companies about long COVID. Finally, we consider that analyzing long COVID from a business and commercial perspective is a novel point of view and complementary to health sciences.

## Figures and Tables

**Table 1:** Research items.

Variable	Item	Question	Source
Purchase behavior	BI1	I purchase great amounts of products	[25,69]
	BI2	I purchase more than I used too	
	BI3	I purchase additional products to storage them	
	BI4	I expend more money purchasing	
Uncertainty	Unct1	I feel that purchasing involves a high degree of uncertainty	[20]
	Unct2	I feel the uncertainty associated with purchasing is high	
	Unct3	There is a high degree of uncertainty when I make a purchase	
Legitimacy	Leg1	When I purchase, I care about how trustworthy the company is	[3]
	Leg2	When I purchase, I take into consideration if the company is ethical	
	Leg3	When I purchase, I take into consideration if the company has what I need	
	Leg4	When I purchase, I take into consideration if the company behaves in the best possible manner	
	Leg5	I feel identified with the company	
	Leg6	The correct management of the company is important to me	
	Leg7	The fact that the company fulfills the laws and norms is important	
Anxiety	Anx1	I feel anxious	[25,77]
	Anx2	I feel stressed	
	Anx3	I feel nervous	
	Anx4	I feel concerned	
	Anx5	I feel restless	
	Anx6	I feel scared	
	Anx7	I feel afraid	
	Anx8	I feel alarmed	
Emotional regulation	ER1	I purchase things to relax (for example, food, beverages, entertainment, leisure)	[9]

**Table 2:** Results from the factorial analysis.

Variable	Item	Extracted loadings	Cronbach Alpha	Explained Variance
Purchase Behavior	BI1	0.790	0.815	65.821%
	BI2	0.754		
	BI3	0.842		
	BI4	0.557		
Uncertainty	Unct1	0.909	0.949	90.714%
	Unct2	0.985		
	Unct3	0.890		
Legitimacy	Leg1	0.772	0.896	62.578%
	Leg2	0.841		
	Leg3	0.672		
	Leg4	0.860		
	Leg5	0.602		
	Leg6	0.700		
	Leg7	0.787		
Anxiety	Anx1	0.831	0.955	76.178%
	Anx2	0.783		
	Anx3	0.837		
	Anx4	0.809		
	Anx5	0.897		
	Anx6	0.901		
	Anx7	0.896		
	Anx8	0.861		
Emotional regulation	ER1	-	-	-

**Table 3:** ANOVA results: analysis of the differences by group.

Variable	Sum of Squares	gl	Root Mean Square	F	Sig.
Purchase behavior	16.787	2	83.93	10.19	<0.001
Uncertainty	19.076	2	9.53	10.84	<0.001
Legitimacy	9.154	2	4.57	5.08	0.007
Anxiety	36.424	2	18.21	20.53	0.001
Emotional regulation	29.699	2	14.85	4.98	0.007

**Table 4:** Results of the regression analysis by group.

Target	Variable	Beta	T-Value	*p*-Value
**Long-** **COVID**	(Constant)		−6.312	<0.001
	**Uncertainty**	**0.154**	**1.988**	**0.049**
	**Legitimacy**	**−0.144**	**−2.001**	**0.047**
	Anxiety	0.122	1.482	0.14
	**Eomotional regulation**	**0.508**	**7.541**	**<0.001**
**No COVID**	(Constant)		−5.709	<0.001
	**Uncertainty**	**0.237**	**2.547**	**0.012**
	Legitimacy	0.002	0.032	0.974
	**Anxiety**	**0.224**	**2.652**	**0.009**
	**Emotional regulation**	**0.392**	**6.024**	**<0.001**
**Recovered from COVID**	(Constant)		−1.208	0.229
	Uncertainty	0.035	0.337	0.737
	Legitimacy	0.031	0.372	0.711
	**Anxiety**	**0.249**	**2.297**	**0.023**
	**Emotional regulation**	**0.262**	**2.854**	**0.005**

R^2^: Long COVID = 0.425; No COVID-19 = 0.342; Recovered from COVID-19 = 0.207.

## Data Availability

The data presented in this study are available on request from the corresponding author. The data are not publicly available due to participant consent statement.
